# Biting Back: Advances in Fighting Ticks and Understanding Tick-Borne Pathogens

**DOI:** 10.3390/pathogens13010073

**Published:** 2024-01-13

**Authors:** Anastasia Diakou

**Affiliations:** Laboratory of Parasitology and Parasitic Diseases, School of Veterinary Medicine, Faculty of Health Sciences, Aristotle University of Thessaloniki, 54124 Thessaloniki, Greece; diakou@vet.auth.gr

## 1. Introduction

Ticks are blood-feeding arthropods and obligate ectoparasites of virtually all animal species (except fish) and humans. They are mainly classified into two families, i.e., Ixodidae (“hard ticks”) and Argasidae (“soft ticks”), while a third family, Nuttalliellidae, includes only the monospecific genus *Nuttalliella* [1]. The large family of Ixodidae contains most of the veterinary and medically important ticks that display a direct clinical impact on their hosts, e.g., irritation, anaemia, skin lesions, granulomas, and toxicosis (i.e., tick paralysis), but most significantly, they are the vectors of pathogens (i.e., viruses, bacteria, and protozoa), many of zoonotic character, that threaten animal and human health. In fact, ticks are the most important vectors of pathogens; it suffices to note that in the USA, 95% of vector-borne disease cases are tick-borne [2].

In our fast-changing world, various factors are identified to pave the way for the spreading of ticks and the emerging of tick-borne diseases (TBD). These factors include climate and land use change, the increased movement of animals and humans, and several crises, such as economic and humanitarian emergencies. For this reason, the scientific community should stand guard against the risk of TBD and keep a vigilant eye through monitoring and research related to the epidemiology and control of ticks and tick-transmitted pathogens (i.e., tick-borne pathogens, TBP).

This Editorial summarises the information provided in ten articles published in the journal *Pathogens* in the first semester of 2023, which were among the “Editors’ Choice” for the important information they bring to the scientific community.

## 2. Impact of Ticks and TBD

### 2.1. Pathogenesis of Anaemia in Canine Babesiosis: Possible Contribution of Pro-Inflammatory Cytokines and Chemokines—A Review—By Zygner et al. [3]

The pathogenesis, clinical presentation, diagnosis, and treatment of TBD are wide topics that warrant consistent research and better understanding. In their article, Zygner et al. [3] analyse the literature related to the mechanisms of anaemia induced by the intraerythrocytic, tick-transmitted protozoan parasites of the genus *Babesia*. Anaemia in babesiosis is one of the most severe implications of the infection and, in some cases, a prognostic marker for the survival of the infected dog. However, anaemia is not induced by the parasite per se, and the sheer destruction of red blood cells (RBC) caused by the merozoite development is considered minor [4]. Anaemia is rather the result of the immune response that leads both to the elimination of parasites and erythrocyte destruction.

Several immunological mechanisms contribute to immune-mediated anaemia in babesiosis. Phagocytosis of RBC by macrophages and neutrophils is activated by cytokines such as TNF-a, IFN-γ, CCL2, and CSF-2 in the presence of *Babesia* infection, resulting in excessive splenic and peripheral red blood cell elimination. Similarly, alterations in the levels of IL-10, IL-8, IL-18, IL-12, and CXCL1-like chemokines are likely associated with phagocytic anaemia in canine babesiosis.

Another mechanism leading to anaemia in canine babesiosis is oxidative damage to RBC caused by the release of reactive oxygen species that act directly or, probably, also through the activation of platelets. The effect of oxidative damage is more clearly evidenced in *Babesia gibsoni* than in other *Babesia* species infections, although the stage and severity of disease (complicated vs. uncomplicated) may be critical in these observations. Several cytokines have a critical role in oxidative damage to erythrocytes, with TNF-a being the most important. Overall, oxidative damage is induced by many different and intertwined paths, e.g., activation of monocytes, stimulation of neutrophils, phagocytosis, and relative balance in the levels of a large set of cytokines. The mechanisms of phagocytosis and oxidative damage are also activated by anti-erythrocyte antibodies that induce opsonization of RBC and subsequent complement activation. Furthermore, antibodies against RBC result in a form of autoimmune anaemia in dogs infected with some *Babesia* species.

The role of antibody-dependent cellular cytotoxicity and the complement system has not been extensively studied in canine babesiosis, but evidence from similar parasitic infections in other hosts indicates that these mechanisms may participate in the development of anaemia or the elimination of the parasite. Similarly, splenomegaly and alterations in the structure of the spleen parenchyma, as well as splenic retention of RBC, have been demonstrated in babesiosis. Nevertheless, although the associated mechanisms have been studied in other animals, in canine babesiosis, the related surveys are scant. Sequestration of erythrocytes in micro-vessels during babesiosis is yet another anaemia-associated mechanism, triggered by the presence of specific proteins on the infected erythrocyte membrane. Sequestration (and thus elimination from the circulation) of infected RBC could be the reason for the observed “low parasitaemia” in cases of babesiosis-induced anaemia.

The authors throughout the article [3] emphasise numerous analogies between canine babesiosis and other haemoprotozoan infections in mammals and, particularly, human malaria, underlining the importance of research on canine babesiosis as a model that can offer insights into human malaria, which is a major human vector-borne disease.

### 2.2. Neurologic Features of Lyme Disease That May Present to a Rheumatologist—By Govil et al. [5]

Lyme disease (LD) is one of the most widespread and important TBDs, caused by *Borrelia burgdorferi* (*s.l*.), displaying a variety of clinical manifestations, e.g., skin rash (*Erythema migrans*), neurological signs, and arthralgia [6]. Due to the latter manifestation, rheumatologists are often called upon to diagnose LD. In their article, Govil et al. [5] aim to alert rheumatologists and medical doctors in general about the neurological signs that should incite them to include LD in differential diagnosis. Doctors should keep in mind that LD cases display seasonality and arise mostly during the warm months of the year. The authors emphasise the significance of obtaining specific information from the patient’s medical history, particularly regarding incidents of tick bites and the presence of skin lesions resembling erythema migrans. It is important to note that skin manifestations in LD may not always exhibit a typical appearance. Additionally, unlike arthralgias, these manifestations are expected mostly in the early stages of infection and may not be present during examination by the rheumatologist.

The neurological manifestation of LD is very prevalent [7]. The most common implications include cranial nerve VII palsy, aseptic meningitis syndrome, and acute painful radiculoneuritis. Mild chronic encephalopathy is the most frequent neurological manifestation in the late stage of the disease, appearing with amnesia, sleep disorders, fatigue, and depression. Some less common manifestations observed in the late neurological LD include subtle encephalopathy, encephalomyelitis, and neuropathies (e.g., mononeuropathy multiplex and sensory axonal peripheral neuropathy). It is thus essential to consider LD in the differential diagnosis in all cases where compatible clinical signs are present. Two-tiered tests (ELISA coupled with Western immunoblot or with a second ELISA, as in the most recently improved diagnostic procedure) are widely applied for the diagnosis of LB, but a positive result does not always correspond to an active infection and could be due to simple exposure to *B. burgdorferi*. Conversely, a positive PCR in a blood or CSF sample is a strong indication of an active infection, while detection of *B. burgdorferi* DNA in the synovial fluid would confirm LD as the causative agent of arthritis.

The authors conclude that a doctor’s preparedness (possibility for interdisciplinary management with a collaborating laboratory and neurologist for prompt referral) would ensure the most favourable outcome for the patients [5].

### 2.3. Borrelia miyamotoi: A Comprehensive Review—By Cleveland et al. [8]

One of the most recently described TBDs is *Borrelia miyamotoi* disease (BMD). Interestingly, although *B. miyamotoi* phylogenetically belongs to the relapsing fever (RF) group of spirochaetes, which includes *Borrelia* species transmitted by Argasidae ticks and lice, it is also transmitted by Ixodidae ticks, also vectoring LD [9]. For this reason, it is characterised as a “hard tick-borne RF *Borrelia*”. Cleveland et al. [8] in their Review Article present the pathogenicity, ecological maintenance, transmission, and genetic characteristics of the causative agent and provide the latest information about the prevalence and diagnostic approaches of this emerging TBD.

In general, BMD in immunocompetent patients remains subclinical or develops as a mild febrile disease with nausea, exhaustion, myalgias, and arthralgias. Relapses of the fever may occur in some cases due to antigenic alterations of the microorganism. However, in immunocompromised patients, BMD can be life-threatening, presenting neurological signs due to meningoencephalitis. Microscopic detection of *Borrelia* in the blood or CSF is a diagnostic approach but has a low sensitivity, even in the acute phase of the disease, and it cannot distinguish the species of spirochaete involved. PCR, on the other hand, can be more sensitive and specific, depending on the biological sample examined and the timing. A recently developed semi-multiplex real-time PCR is promising for a prompt and accurate aetiological diagnosis of BMD and RF spirochaetes. Detection of specific antibodies by ELISA or Western blot is an additional diagnostic tool for BMD; however, the results depend on the antigens selected, time of examination, and immunocompetence of the patient, while cross-reactions, leading to misdiagnosis (especially with LD), seem common. Overall, microscopy combined with PCR is more reliable for diagnosing acute disease.

Most clinical cases have been diagnosed in Russia, a fact that could be attributed to the possibly increased vectorial capacity of *I. persulcatus*, which is present in the area. Cases have also been reported in the USA and France, while a few cases were diagnosed in other areas of Asia and Europe. Retrospective seroprevalence studies show that the infection ranges from 1.3% in healthy individuals to 11.9% in patients suspected of TBD.

*Borrelia miyamotoi* can be transmitted both horizontally, i.e., from an infected tick to a vertebrate host and vice versa, and vertically, i.e., transovarially, from an infected female tick to the offspring. Two additional ways of transmission are also suggested, i.e., via co-feeding between ticks and vertically in mammals, from pregnant females to the offspring. There is evidence that the infection of a mammal can take place during the first 24 h of a tick blood meal. The ticks considered vectors of the disease, depending on the area of the world, are *Ixodes persulcatus* in Europe and Asia, *I. ovatus*, *I. nipponensis*, and *I. pavoloskyi* in Asia, *I. ricinus* in Europe, and *I. scapularis* in the USA and Canada. Other widely distributed ticks, e.g., *Dermacentor reticulatus* and *Haemaphysalis* spp., have also been found positive for *B. miyamotoi,* but their vectorial role is yet to be clarified.

Small vertebrates, i.e., rodents, mainly of the genera *Peromyscus*, *Apodemus*, and *Myodes*, are considered reservoirs of *B. miyamotoi*. Importantly, infected rodents have been found in both urban and rural settings, suggesting that there is an increased risk for human infection [10]. The persistence and maintenance mechanisms of *B. miyamotoi* in reservoir rodents are yet to be defined, but there is evidence that they are independent of rodent age, month of infection, infection presence, and rodent population density. Larger animals and birds have occasionally been found positive too, while observations in white-tailed deer imply that this animal may also be a reservoir of the microorganism.

Genetic analysis of *B. miyamotoi* isolates revealed three geographically based lineages: the Asian, the European, and the North American. Genetic variations may be related to infectivity and pathogenicity, and future studies may shed light on this. Cleveland et al. [8] stress the importance of raising awareness about BMD both in the scientific community and the public and highlight the need for the development of standardised methods that will ensure prompt and accurate diagnosis of the disease.

## 3. Epidemiology of Ticks and TBD

### 3.1. Human Borrelia miyamotoi Infection in North America—By Burde et al. [11]

Only about a month after the paper of Cleveland et al. [8] about *B. miyamotoi*, a Review Article titled “Human *Borrelia miyamotoi* Infection in North America” saw the light of publicity, adding detailed information about the epidemiological situation in the USA and Canada [11]. This article [11] sets the background of history and knowledge about *B. miyamotoi* and the associated disease through a comprehensive review of the literature before reporting the occurrence of the disease in the northern Americas. The tick *I. scapularis* is found widely infected with *B. miyamotoi* in the northeastern, northern, midwestern, and western USA. *Ixodes pacificus* also carries the pathogen in western areas of the country. However, *B. burgdorferi* remains far more prevalent in the USA than *B. miyamotoi*, both in ticks and in humans, based on seroepidemiological studies. In Canada, *B. miyamotoi* has been found in *Ixodes* ticks in all provinces but not in Newfoundland and Labrador. In a serological investigation of blood donors from Manitoba, it was found that 3% of individuals have been exposed to the pathogen. An important epidemiological fact is an increase in the tick vector population in Canada due to climate change, foreshadowing an analogous increase in human *B. miyamotoi* infection [12].

*Borrelia miyamotoi* infection is reportable only in a few states but not nationally in the USA, and it is likely that there are several missed diagnoses, as patients do not seek medical care, an aetiological diagnosis is not achieved, or it is not reported to the public health authorities. There are four *B. miyamotoi* meningoencephalitis cases reported in the USA, while no clinical case has been reported from Canada thus far. The authors suggest that the complete impact of *B. miyamotoi* on health is still to be fully understood [11].

### 3.2. Seroprevalence of Tick-Borne Encephalitis (TBE) Virus Antibodies in Wild Rodents from Two Natural TBE Foci in Bavaria, Germany—By Brandenburg et al. [13]

While *B. miyamotoi* discussed above [11] is an emerging disease tick-borne encephalitis (TBE) is one of the most important TBD in areas of Asia and Europe. TBE has been a notifiable disease in the European Union since 2012. *Ixodes ricinus* and *I. persulcatus* are the vectors of the causative agent (a *Flavivirus*), while rodents are the main vertebrate reservoirs [14]. Brandenburg et al. [13] studied the TBE virus infection trends by serological examination in rodents from two well-studied natural foci of TBE located in the most affected TBE districts of Germany. In fact, the TBE virus has been detected throughout the years since 2009 in ticks collected from these areas. In the study of Brandenburg et al. [14], rodents of the species *Clethrionomys glareolus* and *Apodemus flavicollis* were examined monthly, for 8 months per year (March to October), for four years (2019–2022), in a “capture-mark-release-recapture” protocol. The seroprevalence was determined in blood serum and thoracic lavage samples by indirect immunofluorescence assay (IIFA) and confirmed by a serum neutralisation test (SNT). According to the results, the seroprevalence was higher in *C. glareolus* than in *A. flavicollis* (19.4% vs. 10.5%, respectively). Interestingly, males and adults with *C. glareolus* were more prevalently seropositive than females and juveniles, respectively. A significant conclusion of the study was that the likelihood of rodents getting infected is more influenced by individual factors like species, age, and sex than by external abiotic and biotic factors such as the study site, the year, or the season. The study of Brandenburg et al. [14] was the first to examine reservoir animals for antibody detection over four years in Germany. The authors suggest that more studies on TBE circulation dynamics are necessary to enhance comprehension of the epidemiology and thus infection risk for humans with this important TBP [13].

### 3.3. Isolate of Theileria orientalis Ikeda Is Not Transstadially Transmitted to Cattle by Rhipicephalus microplus—By Onzere et al. [15]

A fundamental factor in TBP circulation is the vector distribution [16]. *Theileria orientalis* is a tick-borne haemoprotozoon affecting cattle, causing anaemia, abortion, weakness, weight gain reduction, impaired production of milk weight gain, and occasionally a fatal outcome. Of the classified genotypes, *T. orientalis* Ikeda is considered the most pathogenic [17]. *Theileria orientalis* is transmitted mainly by *Haemaphysalis longicornis* [18], a tick that is present in Asia and Australia and has been recently introduced to the USA [19]. The aim of the study conducted by Onzere et al. [15] was to determine whether *Rhipicephalus microplus* is also a competent vector of a *Theileria orientalis* Ikeda isolate circulating in the USA. It is worth noting that *R. microplus* is a vector of other Apicomplexa parasites (*Theileria equi*, *Babesia caballi*, and *Babesia bovis*) and is still encountered in some locations of the USA, despite its eradication from most areas. In this study, *R*. *microplus* larvae were applied to feed on a splenectomised, *T. orientalis* Ikeda-infected calf and then applied as adults to two uninfected, splenectomised calves. These calves were monitored daily by PCR and cytology for *T. orientalis* infection, and the final assessment of any transmission took place 60 days later. All examinations were negative for *T. orientalis*. Furthermore, *T. orientalis* was not detected in the salivary glands of the adult ticks 4–5 days after their attachment to the naïve calves or in their offspring (larvae). According to these results, it is suggested that *R. microplus* is not a competent vector of the U.S.A. in the particular *T. orientalis* Ikeda isolate. This is mainly attributed by the authors [19] to the *R. microplus* life cycle as a one-host tick, given that transstadial transfer is the main way of pathogen transmission for most TBP, as is also the case for the principal vector of *T. orientalis*, i.e., *H. longicornis*, which is a three-host tick. Admittedly, there are still many aspects of *R. microplus* and other ticks’ vectorial role to be further clarified against the complex host-vector-pathogen interactions. Accordingly, the authors suggest that continuous studies and surveillance are necessary for monitoring any spreading of TBP [15].

### 3.4. Effective Methods of Estimation of Pathogen Prevalence in Pooled Ticks—By Fracasso et al. [20]

Ticks and TBP become more prevalent and spread in new areas of the world. As a result, the epidemiology of TBD grows more complex and becomes the centre of intensive research. Therefore, it is pivotal to establish well-validated methods for monitoring and surveillance of TBP epidemiological trends. The article by Fracasso et al. [20] provides insights on two important pillars of TBP epidemiological research: (a) the tick-pooling strategies applied before pathogen detection analyses, and (b) the statistical methodologies applied for the determination of pathogen prevalence in tick populations. Furthermore, to provide a practical comparison between the statistical methods discussed, the authors analyse data about infection prevalence collected in a previously published study on ticks from northeastern Italy [21]. The final number of 191 papers retrieved from PubMed were analysed in this study.

Of the different pool examination strategies applied, the variable pool size, based on the sampling site or host species, seems to provide more accurate levels of infection prevalence compared to the fixed pool size. The authors highlight the need for an accurate description of the pooling criteria so that comparisons to the results of other studies have a realistic basis and also stress the importance of not mixing different life stages in the same pool, as this would be confounding regarding the biological traits of infection and not easy to compare between studies. In practical terms, the authors propose, when designing a survey, to run preliminary tests that will help determine the suitable pooling strategy, which will reasonably balance test sensitivity, workload, and cost.

Regarding the statistical analysis, three main methods were used in the retrieved papers: the pool positivity rate (PPR, the ratio of positive pools to the total number of pools tested), the minimum infection rate (MIR, the ratio of positive pool numbers to the total number of specimens tested), and the maximum-likelihood estimate of pooled prevalence (EPP, the infection rate most likely observed given the test results and an assumed probabilistic model). According to the estimations of the study [20], EPP should be preferred over the others, given its accuracy and flexibility in accounting for spatiotemporal confounding effects. Nevertheless, the authors suggest using, when possible, both the EPP and MIR methods so that comparison between studies is feasible, given that MIR is the most widely used method for analysis of results obtained from pooled samples [20].

## 4. Control of Ticks and TBD

### 4.1. Advances in Babesia Vaccine Development: An Overview—By Jerzak et al. [22]

Research focused on the control and elimination of TBD is a fundamental part of the related scientific activity. Three papers associated with tick or TBP control were among the “Editors’ Choice” of the journal *Pathogens* in the first half of 2023. These papers are related to vaccines and tick control.

The tick-transmitted protozoan parasites of the genus *Babesia* are agents of severe animal disease concerning dogs, cats, swine, goats, sheep, horses, and cattle, causing significant economic losses in animal production [23]. Some *Babesia* species can also infect humans, with *Babesia microti* in the USA, Japan, and north-eastern Eurasia and *B. divergens* in Europe having the highest impact among them [24]. It is thus expected that research focused on vaccine development against babesiosis will be intense. Jerzak et al. [22] published a comprehensive Review Article on the advances in *Babesia* vaccine development.

The attempts for vaccine development against *B. microti* in humans include whole parasite vaccines and subunit vaccines. In the first case, inactivated or attenuated whole parasites proved to be quite safe and efficient in protecting animals in murine models against parasitaemia and disease development. However, such vaccines exhibit some major disadvantages, including difficulties in maintaining the parasite cultures, the risk of contamination with other microorganisms, the need for a strong adjuvant for enhanced immunogenicity, and the presence of RBC membranes in the vaccine that could represent an antigenic stimulation for the recipient. Thus, the efforts in further development of whole-parasite vaccines are focused on the reduction or elimination of RBC, adjuvant use, and further development of a polyvalent vaccine against both *B. microti* and *B. divergens*, i.e., the major species causing human babesiosis.

In the case of subunit vaccines, proteins specific to the merozoite stage, which is the stage with higher exposure to antibodies than others, are used. In this context, surface and internal antigens, the moving junction structure responsible for RBC invasion, and the parasite-induced cytoadherence of parasitised RBC are used to induce antibody production. Such vaccines display many advantages (e.g., absence of live parasites, stability), but also the limitation of different isolates, genetic variations, a high diversity of seroreactive antigens, and the need for a strong adjuvant. In the effort for further development of protein-based subunit vaccines, new proteins based on genomic expression libraries are tested, and novel methods, e.g., transfection, and trained immunity using BCG vaccines are attempted.

Attenuated vaccines developed against cattle babesiosis have proven effective, and the proposed strategy is to vaccinate calves under one year of age in enzootic countries. However, there are significant challenges in the production of these vaccines, such as the risk of other pathogen transmission, the risk of reversion of virulence, and the requirement of a cold chain for storage, which is often difficult to ensure in tropical enzootic areas of the world. The efforts for the development of better vaccines against *B. bovis* focus on the use of various antigenic proteins to be targeted. Among these are proteins involved in parasite binding to the erythrocyte membrane and in the invasion process (e.g., merozoite surface antigens 1 and 2, ribosomal phosphoprotein P0, and rhoptry-associated protein 1). Furthermore, the transfection process is under investigation as a powerful tool in the fight against cattle babesiosis.

In this article, Jerzak et al. [22] explore the wide area of vaccine development attempts against human and animal babesiosis with classical and novel methodologies, showing the significant progress achieved and at the same time highlighting the need for ongoing research in this field.

### 4.2. Does Experimental Reduction of Blacklegged Tick (Ixodes scapularis) Abundance Reduce Lyme Disease Incidence?—By Ostfeld and Keesing [25]

Tick population reduction is an essential part of TBD control; it can be attempted indirectly, through interventions to the habitat (vegetation management, host control), and directly, using chemical or biological acaricides [26]. However, although there is a great body of research on the efficacy of different tick control treatments, the actual effect of such attempts on human exposure to TBD is rarely evaluated; it is rather arbitrarily assumed [25]. In their Review Article, Ostfeld and Keesing [25] investigate this drawback in the scientific literature, specifically the direct control of *Ixodes scapularis*, the vector of LD, and discuss the cause of discrepancies in the conclusions of different studies. The main goal of this approach, as the authors describe it, is to evaluate the strength of the evidence in the literature that experimental reduction of the *I. scapularis* population diminishes human contact with *B. burgdorferi*.

Interestingly, the results of this literature review indicate that although tick reduction was considerable and promising when evaluated in small-scale field trials, in large-scale studies, or when critical study-design parameters are included (e.g., randomisation, placebo controls, and blinding), the efficacy of the same acaricides was lower or null. Importantly, only the latter studies included investigations of human encounters with ticks and the occurrence of TBD. Although the reasons for these discrepancies are not clear, they raise concerns about the actual relevance of the studies and reveal the need to set standards in designing related research protocols. Furthermore, the interpretation of results in such studies should be cautious, especially given that many factors influence the outcomes. The authors stress the importance of continued development and assessment of acaricidal products but also emphasise the need for rigorous study designs that will also evaluate effects on TBD epidemiology. As the inclusion of parameters such as randomisation, placebo controls, blinding, and monitoring humans significantly augments the cost of research, funding for this field of study should be increased [25]. This would represent an important investment and effective relocation of resources because reliable results would indicate the most effective and realistic path to TBD control.

### 4.3. The Role of Parasitoid Wasps, Ixodiphagus spp. (Hymenoptera: Encyrtidae), in Tick Control—By Ramos et al. [27]

Other than acaricides, biological control of ticks is of particular interest. The emergence of tick resistance to acaricide drugs, as well as concerns about the consequences of chemical use for the environment and organisms, give prominence to the exploration and application of alternative methods for tick control [28]. The wasps of the genus *Ixodiphagus* (Hymenoptera, Encyrtidae) are parasitoids of ixodid ticks [29], and the perspective of their use as biological control of ticks has triggered scientific interest and several attempts to evaluate their efficacy in diminishing tick populations. In their Review Article, Ramos et al. [27] investigate the role of *Ixodiphagus* spp. in tick control, as this is described in the scientific literature. The authors initially present details of the life cycle and biological characteristics of *Ixodiphagus* spp., some of which are decisive in their potential role as a tick control tool. These biological characteristics include attractive stimuli for female *Ixodiphagus* spp. concerning tick or vertebrate host species, the need for blood meal in ticks for the development of wasp larvae, the seasonality of development, and overwintering capability. Despite the lethal effect of these parasitoids on ticks, the perspective of their use as biological control is still weak. In fact, of all the attempts to use these wasps, only a study in Kenya, monitoring the effect of *Ixodiphagus hookeri* on the tick infestation of 10 cattle, showed promising results [30]. The reasons identified by Ramos et al. [27] for the mostly unsuccessful use of *Ixodiphagus* spp. are related to the parasitoids’ biology and the design of the studies. More specifically, the climatic conditions, the affiliation of the wasp species with the target tick species, the density of ticks and vertebrate hosts in application, and the wasp-releasing strategies (i.e., single, multiple, and the wasp’s life stage) are parameters that merit careful study. Furthermore, planning long-term follow-up monitoring of any effect is essential in order to evaluate the sustainability and practical value of this control method. Taking the above into consideration, the authors conclude that the use of parasitoids is difficult to apply as an alternative tick control strategy.

## 5. Closing Remarks

Among the articles chosen by the Editors of Pathogens in the first semester of 2023 as of particular interest, ten were related to ticks and TBD. These articles addressed matters concerning the impact and clinical importance of TBD, the epidemiology of TBD, and control strategies for both ticks and TBD. Eight out of the ten articles were Review or Opinion Articles, indicating the need for analysis, evaluation, and reflection on the rapidly increasing knowledge. Indeed, synthesising a comprehensive review of the literature, especially in fields of intense research and abundant result production, is crucial as it helps the scientific community stay up-to-date with the overwhelming amount of new information. Hopefully, this Editorial efficiently summarises the main points of all ten articles, further supporting the reader in keeping track of the latest knowledge on ticks and TBD.

## References

[1] Nava S., Guglielmone A.A., Mangold A.J. (2009). An overview of systematics and evolution of ticks. Front. Biosci. Landmark Ed..

[2] Rochlin I., Toledo A. (2020). Emerging tick-borne pathogens of public health importance: A mini-review. J. Med. Microbiol..

[3] Zygner W., Gójska-Zygner O., Norbury L.J. (2023). Pathogenesis of Anemia in Canine Babesiosis: Possible Contribution of Pro-Inflammatory Cytokines and Chemokines—A Review. Pathogens.

[4] Kelly P., Köster L.S., Lobetti R.G. (2015). Canine babesiosis: A perspective on clinical complications, biomarkers, and treatment. Vet. Med. Res. Rep..

[5] Govil S., Capitle E., Lacqua A., Khianey R., Coyle P.K., Schutzer S.E. (2023). Common Neurologic Features of Lyme Disease That May Present to a Rheumatologist. Pathogens.

[6] Marques A.R., Strle F., Wormser G.P. (2021). Comparison of Lyme Disease in the United States and Europe. Emerg. Infect. Dis..

[7] Johnson K.O., Nelder M.P., Russell C., Li Y., Badiani T., Sander B., Sider D., Patel S.N. (2018). Clinical manifestations of reported Lyme disease cases in Ontario, Canada: 2005–2014. PLoS ONE.

[8] Cleveland D.W., Anderson C.C., Brissette C.A. (2023). *Borrelia miyamotoi*: A Comprehensive Review. Pathogens.

[9] Fukunaga M., Takahashi Y., Tsuruta Y., Matsushita O., Ralph D., McClelland M., Nakao M. (1995). Genetic and Phenotypic Analysis of *Borrelia miyamotoi* Sp. Nov., Isolated from the Ixodid Tick *Ixodes persulcatus*, the Vector for Lyme Disease in Japan. Int. J. Syst. Bacteriol..

[10] Sormunen J.J., Andersson T., Aspi J., Bäck J., Cederberg T., Haavisto N., Halonen H., Hänninen J., Inkinen J., Kulha N. (2020). Monitoring of Ticks and Tick-Borne Pathogens through a Nationwide Research Station Network in Finland. Ticks Tick Borne Dis..

[11] Burde J., Bloch E.M., Kelly J.R., Krause P.J. (2023). Human *Borrelia miyamotoi* Infection in North America. Pathogens.

[12] Ogden N.H., Ben Beard C., Ginsberg H.S., Tsao J.I. (2020). Possible effects of climate change on Ixodid ticks and the pathogens they transmit: Predictions and observations. J. Med. Èntomol..

[13] Brandenburg P.J., Obiegala A., Schmuck H.M., Dobler G., Chitimia-Dobler L., Pfeffer M. (2023). Seroprevalence of Tick-Borne Encephalitis (TBE) Virus Antibodies in Wild Rodents from Two Natural TBE Foci in Bavaria, Germany. Pathogens.

[14] Mansfield K.L., Johnson N., Phipps L.P., Stephenson J.R., Fooks A.R., Solomon T. (2009). Tick-borne encephalitis virus—A review of an emerging zoonosis. J. Gen. Virol..

[15] Onzere C.K., Herndon D.R., Hassan A., Oyen K., Poh K.C., Scoles G.A., Fry L.M. (2023). A U.S. Isolate of *Theileria orientalis* Ikeda Is Not Transstadially Transmitted to Cattle by *Rhipicephalus microplus*. Pathogens.

[16] Alkishe A., Raghavan R.K., Peterson A.T. (2021). Likely Geographic Distributional Shifts among Medically Important Tick Species and Tick-Associated Diseases under Climate Change in North America: A Review. Insects.

[17] Bogema D.R., Micallef M.L., Liu M., Padula M.P., Djordjevic S.P., Darling A.E., Jenkins C. (2018). Analysis of *Theileria orientalis* draft genome sequences reveals potential species-level divergence of the Ikeda, Chitose and Buffeli genotypes. BMC Genom..

[18] Watts J.G., Playford M.C., Hickey K.L. (2016). *Theileria orientalis*: A review. N. Z. Vet. J..

[19] Yabsley M.J., Thompson A.T. (2023). *Haemaphysalis longicornis* (Asian longhorned tick). Trends Parasitol..

[20] Fracasso G., Grillini M., Grassi L., Gradoni F., Rold G.D., Bertola M. (2023). Effective Methods of Estimation of Pathogen Prevalence in Pooled Ticks. Pathogens.

[21] Bertola M., Montarsi F., Obber F., Da Rold G., Carlin S., Toniolo F., Porcellato E., Falcaro C., Mondardini V., Ormelli S. (2021). Occurrence and identification of *Ixodes ricinus* borne pathogens in Northeastern Italy. Pathogens.

[22] Jerzak M., Gandurski A., Tokaj M., Stachera W., Szuba M., Dybicz M. (2023). Advances in *Babesia* Vaccine Development: An Overview. Pathogens.

[23] Homer M.J., Aguilar-Delfin I., Telford S.R., Krause P.J., Persing D.H. (2000). Babesiosis. Clin. Microbiol. Rev..

[24] Westblade L.F., Simon M.S., Mathison B.A., Kirkman L.A. (2017). *Babesia microti*: From Mice to Ticks to an Increasing Number of Highly Susceptible Humans. J. Clin. Microbiol..

[25] Ostfeld R.S., Keesing F. (2023). Does Experimental Reduction of Blacklegged Tick (*Ixodes scapularis*) Abundance Reduce Lyme Disease Incidence?. Pathogens.

[26] Willadsen P. (2006). Tick control: Thoughts on a research agenda. Vet. Parasitol..

[27] Ramos RA N., de Macedo L.O., Bezerra-Santos M.A., de Carvalho G.A., Verocai G.G., Otranto D. (2023). The Role of Parasitoid Wasps, *Ixodiphagus* spp. (Hymenoptera: Encyrtidae), in Tick Control. Pathogens.

[28] Bonatte P., Barros J.C., Maciel W.G., Garcia M.V., Higa L.O.S., Andreotti R. (2022). Control strategies for the tick *Rhipicephalus microplus* (Canestrini, 1888) on cattle: Economic evaluation and report of a multidrug-resistant strain. Acta Parasitol..

[29] Collatz J., Selzer P., Fuhrmann A., Oehme R.M., Mackenstedt U., Kahl O., Steidle J.L.M. (2011). A hidden beneficial: Biology of the tick-wasp *Ixodiphagus hookeri* in Germany. J. Appl. Entomol..

[30] Mwangi E.N., Hassan S.M., Kaaya G.P., Essuman S. (1997). The impact of *Ixodiphagus hookeri*, a tick parasitoid, on *Amblyomma variegatum* (Acari: Ixodidae) in a field trial in Kenya. Exp. Appl. Acarol..

